# Quick adaptation of the organisation of general practices during the COVID-19 pandemic in the Netherlands

**DOI:** 10.1186/s12875-023-02114-5

**Published:** 2023-08-31

**Authors:** Peter P. Groenewegen, Maria van den Muijsenbergh, Ronald Batenburg, Esther Van Poel, Stijn van den Broek, Pierre Vanden Bussche, Sara Willems

**Affiliations:** 1 Nivel (Netherlands Institute for Health Services Research), Utrecht, the Netherlands; 2 Department of Primary Care and Community Care, Radboud University, Nijmegen, The Netherlands; 3 Radboud University Nijmegen, Nijmegen, The Netherlands; 4 Department of Public Health and Primary Care, Quality and Safety, Ghent, Belgium; 5 Nederlands Huisartsen Genootschap, Utrecht, The Netherlands

**Keywords:** The Netherlands, General practice, COVID-19 pandemic, Practice organisation, Primary care, Quality of care, Infectious disease, PRICOV-19 study

## Abstract

**Background:**

General practices have adapted the practice organisation to the circumstances of the COVID-19 pandemic. In this article we describe several adjustments in general practices in the field of patient flow management, appointments, triage, referral and infection prevention. We also examined how practices relate to the policy of the government and of the professional organisations during the pandemic.

**Methods:**

A cross-sectional online survey was conducted among a sample of 893 general practitioners (GPs) during February and March 2021. The response rate was 17%. Because the questionnaire concerns practices and not individual GPs, one practice owner per practice received an invitation with a link to the online questionnaire. One reminder has been sent.

**Results:**

General practices adapted their organisation during the corona pandemic, partly based on information and advice from their professional organisations. The adjustments were necessary to ensure that patient care continued as much and as safely as possible, often remotely. The use of video consultations quickly increased from 6% to 65% of the practices. The cooperation with neighbouring practices improved and practices felt supported by the professional organisations.

**Conclusions:**

The pandemic itself, remote care and stricter patient flow management have put pressure on the quality of care and patient safety. The accessibility of the practices was sometimes limited. In the perception of patients, this was stronger than in reality.

## Background

The corona pandemic has (had) major consequences for general practitioners (GPs). Due to the limited care capacity in hospitals, they received the majority of patients with possible COVID-19 complaints [1–5]. The enormously increased demand for care from patients with COVID-19 - for diagnostics, guidance, treatment, referral or palliative care - made it from the start of the pandemic necessary to reorganise practice in such a way that the safety of employees and patients was guaranteed and that other patients could also continue to receive the necessary care.

Because the role of primary care differs between European countries, the corona pandemic offers opportunities for cross-country learning [6]. This was the reason to set up the PRICOV-19 study, a pan-European questionnaire survey among GPs into the consequences of the pandemic for their functioning and for the organisation of their practice. In this article we present some of the Dutch results of this research. First we will briefly sketch the context of Dutch general practice and of the pandemic in the Netherlands and the role of general practice during the pandemic.

### Context of Dutch general practice

The Dutch health care system is based on social insurance funding, in traditionally corporatist governance, with a relatively distant role for the central government [7]. The insurance system is privately organised, but publicly guaranteed. Every citizen is obliged to take out health insurance with one of the competing insurance companies. Citizens pay a fixed, community rated premium and depending on their income, can receive compensation. Insurance companies are obliged to contract the necessary care to serve their insured. They can selectively contract certain providers based on price and quality of care. Practically, all insurance companies have contracts with all GPs and follow in most respects the insurer with the highest regional share of insured.

In 2021, 13,492 GPs were actively employed the Netherlands, working in 4,999 general practices. Taking part-time working into account, the provider-population ratio was 5.3 full-time equivalents (fte) GP per 10,000 inhabitants. General practices in the Netherlands are relatively small:

- 35% of the practices are single-handed (i.e. staffed by one GP);
- 42% of the practices are staffed by two GPs, and.
- 23% of the practices are staffed by more than two GPs.

The largest category of GPs is self-employed, 51%; 28% works as an employee in a health centre or practice, and 21% works as a locum in different practices [8].

All practices employ practice assistants (they have broader tasks than secretaries, also telephone triage, and clinical support). Nearly all employ practice nurses for patients with a chronic disease, elderly care, and mental health care.

Nearly all Dutch citizens are on the list of a specific practice they can choose freely. Access to specialist care is only after a referral (gatekeeping). Consultations are relatively short; standard booking time is still ten minutes but increasing.

GPs are paid in a mixed system:

- Capitation with differentiation by patient age and patients living in deprived areas;
- Pay per consultation, also telephone, email and teleconsultations;
- Performance payment for some services to be negotiated with the insurance company in the area of referrals, prescribing, and service and accessibility, and for disease management for a number of chronic diseases.

GP care is covered by the basic health care insurance. All insured pay a mandatory deductible, but GP care is exempted from this; however, the deductible applies to drugs prescribed by a GP, laboratory work, and to follow-up care by specialists after a referral.

There are two large professional organisations, the National College of GPs (NHG) and the National Association of GPs (LHV). The first is the scientific association which focuses on the quality and evidence base of GP care, amongst others, through developing guidelines. The second represents the professional and material interests of general practice.

Overall, the Dutch primary care system is seen as a strong system in international perspective. An important problem, however, is fragmentation and coordination, because other primary care providers, such as physiotherapists and occupational therapists, work in their own practices and have their own insurance coverage and payment modalities [9].

### Context of the pandemic in the Netherlands and the role of general practice

At the height of the first wave, May 2020, the number of confirmed COVID-19 cases in the Netherlands was 2,548 per million inhabitants. A relative moderate number, as twelve of the participating countries in the PRICOV-19 study had more confirmed cases. It should be noted that access to testing for the general population was restricted then. As of June 2020, testing was available for all citizens with symptoms [10]. During the three months before the start of the data collection, the number of confirmed cases was decreased to, on average, 329 per million inhabitants. At that time, seven of the participating countries in the PRICOV-19 study had more confirmed cases, but it should be taken into account that the three-month period before data collection in the PRICOV-19 study differed per country. During the first month of the data collection, there was a curfew in force.

Testing and tracing were done by the local public health authorities that act in groups of municipalities. GPs were (and are) the first contact point for patients with health complaints (possibly) related to COVID-19, usually with special consultation hours. GPs had no role in testing and tracing. Vaccination was the responsibility of the local public health authorities, but GPs were responsible for the vaccination of community dwelling patients who were unable to visit the vaccination sites (e.g. because of age-related frailty). GPs were not responsible for writing sickness absence certificates. Tariffs for telephone and teleconsultations by GPs existed before the pandemic and were open to be used. There was extra compensation for GPs for COVID-19 care, set at EUR 10 for each registered patient in their practice and additionally EUR 15 per hour for extra out-of-hours care provided. The insurance companies also stepped in for costs of personal protective equipment [11].

The health insurance companies did not play a role in decision-making around COVID-19 measures, although they were represented in the Regional Committees of the Organisation of Acute Care (Regionale Organisaties voor Acute Zorg in Dutch).

The COVID-19 pandemic has had a strong impact on health care and society in general. GP practices had to adapt their day-to-day organisation to be as much as possible accessible to their patients, while at the same protecting their staff and patients against infections, e.g. by changing the routing of patients or referral to special COVID centres. In this article, we address the question: How have GPs adapted their practice organisation during the pandemic regarding patient flow management, appointments, triage, referral and infection prevention?

The results will provide GPs with mirror information about how their own measures and experiences relate to those in other GP practices, which is both relevant to Dutch GPs to see how their direct colleagues act as well as for foreign GPs to see how their Dutch colleagues on average work during this difficult period.

## Methods

PRICOV-19 is an international questionnaire survey in general practices, set up in 2020 by the Quality and Safety Ghent expertise centre of the Department of Public Health and Primary Care of Ghent University, in collaboration with two networks of the World Organisation of Family Doctors (WONCA), namely the European Society for Quality and Patient Safety in General Practice/Family Medicine (EQuiP) and the European General Practice Research Network (EGPRN), and the European Forum for Primary Care (EFPC) [12]. The Dutch part was carried out by Nivel and the Department of Primary care at Radboudumc.

In the Netherlands, two samples were taken from the practices within the Nivel GP registry (https://www.nivel.nl/en/beroepenregistraties-de-gezondheidszorg/healthcare-professionals-registries). The first sample consisted of practices that had previously indicated their willingness to participate in research (*n* = 282); the second was randomly drawn from all other practices (*n* = 611). Because the questionnaire concerns practices and not individual GPs, one practice owner per practice received an invitation with a link to the online questionnaire. The PRICOV-19 study used the Research Electronic Data Capture (REDCap) platform to host the survey, send out invitations to the national samples of general/family practices, and securely store the answers from the participants [13]. The data collection took place in February and March 2021. One reminder has been sent.

The questionnaire was developed in several steps by the initiators, discussed with all partners in the research, tested in Flanders (Belgium) and subsequently adapted to the Dutch situation. The questions mainly refer to the situation in the practice at the time of filling out the questionnaire, unless specified differently (some questions ask for the situation since or before the start of the pandemic. It consists of the following components: general data about the practice, patient flows, infection prevention, information processing, cooperation, collegiality and concern for one’s own well-being, and the policy of government and professional organisations. The questionnaire contains both binary (yes/no) answering categories and Likert type answering categories (depending on the type of question: never, rarely, sometimes, usually, always; or strongly disagree, disagree, neutral, agree, strongly agree). Descriptive analyses were performed with Stata 16.1.

## Results

### Response and respondent characteristics

The questionnaire was returned by 208 practices; 155 questionnaires were (almost) fully completed (response based on (almost) fully completed questionnaires is 17%). The responding practices were somewhat larger on average, both in terms of number of patients (average 4300 in our sample against 3940 in the GP registry) and in number of GPs (11% single-handed practices in our sample against 19% nationally). Characteristics of the responding practices are given in Table 1.

**Table 1:** Practice location, practice size in patients and GPs, availability of other staff

	Percentage					N
*Practice location*	Big city	Suburb	Small town	Mixed urban-rural	Rural	
	21.8%	5.5%	22.0%	32.1%	17.6%	165
*Number of GPs*	Single-handed	Two GPs	Three GPs	Four or five GPs	More than five GPs	
	10.2%	36.1%	22.3%	13.3%	18.1%	166
*Number of patients*	Lo − 2399	2400–2799	2800–3699	3700–5999	6000-Hi	
	20.5%	19.3%	19.9%	19.3%	20.5%	166
*Other staff*	Percentage yes					
Trainee GP	43.5%					168
Practice nurse (PN)	Percentage yes					
PN for chronic care	94.6%					168
PN for mental health	94.1%					168
PN for children	39.3%					168
PN for elderly care	39.9%					168
Practice manager	19.6%					168
Dietician	16.7%					168
Physiotherapist	10.1%					168
Podiatrist	7.7%					168
Psychologist	4.2%					168

### Organisational measures for infection prevention

During the corona pandemic, GP practices could take various measures to reduce the risk of infection: reducing the risk of personal contact, adjusting the appointment system and triage, and conducting as many remote consultations as possible (Table 2).

**Table 2:** Organisational measures to control the flow of patients (Percentage of general practices that indicate that they have applied the measure)

	Percentage ‘yes’	Percentage ‘usually/’agree’	Percentage ‘always’/ ’strongly agree’	N
*Appointments and triage*				
When patients want to make an online appointment for this practice, they are shown a message informing them about which complaints they may (not) bring to the practice	71%			79
Patients must state a reason when making an online appointment at the practice	88%			83
Patients must state a reason when making an appointment by phone	95%			159
Patients who made an appointment and where it is unclear whether they pose a risk of infection are called beforehand to verify this		24%	51%	146
In the situation where telephonic triage is performed by someone other than a GP in this practice and he/she needs support when assessing a call, he/she can rely on support from a GP		6%	93%	161
The home visits are organised so that potential COVID-19 patients are seen by one GP at the end of the GP’s round		26%	51%	148
*Adaptations in the practice*				
Performing triage before patients entering this practice	88%			155
Limiting the number of patients in waiting room	94%			155
No longer use of the waiting room	14%			155
Structural changes to the reception area	72%			155
Changing repeat prescription approach in terms of patient attending practice	54%			155
Using e-script or health mail for prescriptions	68%			155
*Administrative documents*				
… these documents are available for pickup in this practice		10%	4%	143
… these documents are sent to the patient by postal mail/are dropped in the patient’s home letterbox		35%	11%	142
… these documents are sent to the patient by regular e-mail.		52%	13%	146
… these documents are made available through a GDPR proof online system		21%	7%	152
*Contact with home care services in case of …*				
Patients are diagnosed with COVID-19		29%	28%	153
Patients are diagnosed with a major infectious disease different from COVID-19 (e.g. HIV, hepatitis carrier status)		34%	16%	147
*Changes in tasks*				
Staff members are more involved in giving information and recommendations to patients contacting the practice by phone		38%	34%	160
Staff members are more involved in giving information or explaining what a caregiver has said to illiterate patients, patients with low health literacy or migrants		34%	21%	148
Staff members are more involved in actively reaching out to patients that might postpone healthcare.		37%	14%	157
Staff members are more involved in the triage of patients (by phone, when entering the practice, …)		34%	45%	158
Since the COVID-19 pandemic, GPs or GP trainees are more involved in actively reaching out to patients that might postpone healthcare		41%	9%	144

In the Netherlands, 81% of the practices used an adapted protocol for telephone consultations and 75% used that protocol regularly or all the time. For more than 70%, the most recent information about how a patient can be referred to a COVID-19 triage post (often one place per region, at a GP out-of-hours location) was immediately available in every consultation room. An important change was video consultations: 6% indicated that they sometimes did this before the pandemic (at most once a week); in our study period 65% used video consultations. Telephone triage also seems to play a more important role, as evidenced by the answers to a question about changes in the tasks of practice staff (not in table).

**Fig. 1:**
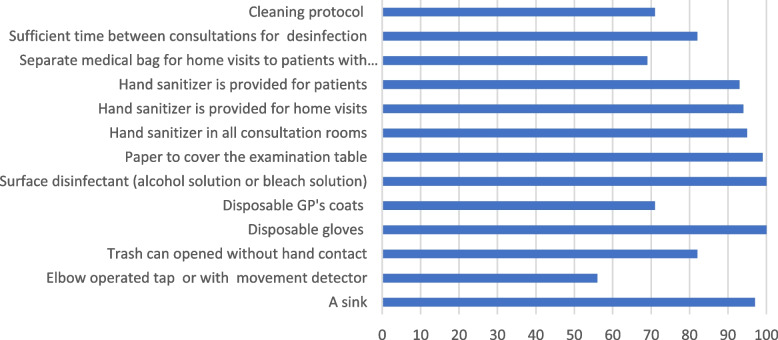
Percentage of GP practices that implement infection prevention measures (N varies between 152 and 154)

In addition to these organisational measures, many practical measures have been taken to prevent infections, such as hand gel in all consultation rooms and cleaning protocols (see Fig. 1).

### Consequences for quality of care and patient safety

Before the pandemic, 19% of responding practices reserved fixed times in the agenda for reviewing new guidelines or literature; at the time of the survey, this was 32%. Comparing the situation before and during the pandemic, a similar difference was also found for discussions on existing, new or amended guidelines – 27% before the pandemic and 68% during the pandemic.

Although attention to new guidelines and collaboration increased, the participants had the impression that the new organisational measures put pressure on patient safety. For example, almost two out of five GPs had the impression that care was sometimes delayed, mainly because patients came late to the practice: 79% had experienced that a patient with a serious condition was seen late (see Table 3).

**Table 3:** Percentage of GP practices indicating that the following patient safety incidents have occurred

	Yes (n)
A patient with a fever caused by an infection other than COVID-19 was seen late due to the fact the COVID-19 protocol was followed which delayed the care	42% (146)
A patient with an urgent condition was seen late because he/she did not come to the practice sooner	79% (149)
A patient with a serious condition was seen late because he/she did not know how to call on a GP	38% (130)
A patient with an urgent condition was seen late because the situation was assessed as non-urgent during the telephonic triage	30% (145)
A patient with an urgent condition other than COVID-19 was assessed incorrectly during the triage procedure	33% (139)

### Absence of practice staff

In nine out of ten practices, staff were temporarily absent during the pandemic (due to illness or quarantine). About half of these cases involved five or more staff members. In nearly a quarter of cases, absenteeism was coped with internally and in nearly one third with the help of neighbouring practices (see Table 4). According to 40% of the practices, a nice side effect was that this promoted cooperation with other practices.

**Table 4:** The way practices coped with absenteeism of staff members (percentages)

	Percentage ‘agree‘	Percentage ‘strongly agree’	N
If staff members in this practice stay home sick, the work can be distributed in such a way that the well-being of colleagues is not compromised	43%	24%	153
If staff members in this practice stay home sick, this practice can count on the help of other PC practices in the neighbourhood	37%	30%	150
The COVID-19 pandemic has promoted cooperation with other PC practices in the neighbourhood	13%	27%	152

### Support from the government, the national institute of public health, and professional organisations

**Fig. 2:**
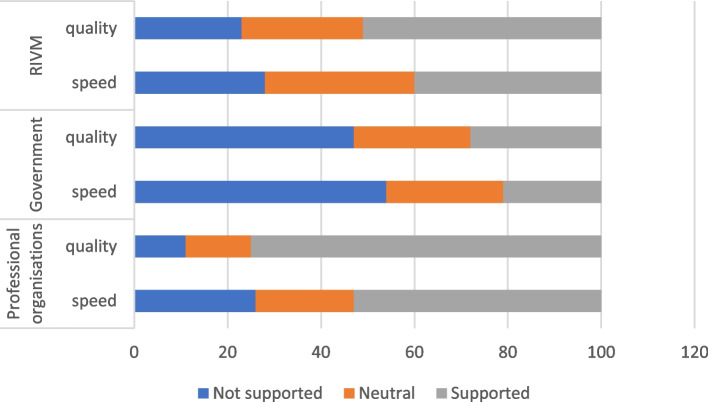
Percentage of general practices that felt supported by professional organisations, the government and National Institute of Public Health (RIVM) (N varies between 150 and 152)

Specific to the Dutch questionnaire, we have added questions about the support that GP practices felt from the Dutch government, the National Institute of Public Health (RIVM) and from the professional organisations. We have distinguished between the perceived quality of the support and the speed with which these organisations supported GP practices Fig. 2 shows that the largest category of practices did not perceive the information provided by the government as supportive. The specific provision of information from the RIVM and especially from the professional organisations was more appreciated.

## Discussion

During the pandemic, digital and remote GP care in the Netherlands was often used, especially through video consultations. Although we do not have information on the use of video consultations before the pandemic, the use at the time of our questionnaire is surprisingly high, which may indicate a rapid increase. Furthermore, the appointment system, triage and patient flow to the practice have been adjusted. The absence of practice staff could often be accommodated internally or with the help of a neighbouring practice; two in five practices found that cooperation with other practices had improved.

The first waves of the pandemic have put pressure on patient safety, among other things, because patients have been reluctant to visit the practice. Reasons may have been that patients did not consider it safe or they did not want to burden the GP unnecessarily [14]. Due to the required infection prevention, patients were less frequently examined physically. The information from the government was usually not experienced as supportive, but that from the Dutch Institute of Public Health RIVM and professional organisations was.

The reported patient safety incidents are another point of interest. These will to some extent reflect the GPs’ perceptions, and perhaps also the safety culture and the extent to which they reflect on and recognise incidents. However, the lack of direct contact with patients in certain phases of the pandemic suggests that many incidents have not (yet) been traced. In this respect it is useful to quote the National College of GPs (NHG) definition of patient safety incidents: ‘Any unintended event (mistake, error, accident, abnormality) that has reached the patient and that has led to, could have caused or (still) could lead to damage to the patient’ [15, 16]. While we do not know the actual health consequences for patients, practices can learn a lot about the safety of their care from a closer analysis of the risk of incidents that could (have) led to these health consequences. For instance, for patients who find it difficult to put their health problems into words, telephone triage is sometimes insufficient, and face-to-face contact is important in order to assess the complaint properly [17, 18].

### Limitations

The response of 17% achieved in the Dutch part of the PROCOV-19 project is reasonable by Dutch standards, but large practices are somewhat overrepresented in the response group. Therefore, the results cannot be generalized to all GP practices in the Netherlands.

Our sample was taken partly from GP practices that had indicated their willingness to participate in research and partly from a random sample of all other practices. The reason to this is that we wanted to keep the number of practices that we approached as low as possible. Dutch GP practices are under high survey pressure, also in a European comparative perspective ([19]; additional file 1 ‘Survey pressure among GPs by country’). This may have introduced bias, as the practices that are interested in research may differ from other practices. However, we were unable to assess this, as it was impossible to identify which practices had responded and which did not. This was a consequence of the design of the data collection of the PRICOV-19 study as a whole, where responding practices could not be identified.

Furthermore, it is a limitation of questionnaire research that we measured subjective estimates of the respondents, for example, regarding patient safety incidents. However, the estimates regarding the postponement of care are confirmed by figures on the number of referrals. A further limitation is that, with a few exceptions, the questionnaire asks about the situation when it was completed (Spring 2021). The point of departure may have influenced the necessary adjustments. The answering categories used in the questionnaire were global and subjective assessment and not exact frequencies. The latter would have required a different research design.

### Implications for practice

The COVID-19 pandemic has demonstrated worldwide the importance of GP care, but has also shown where improvements can be made [20–22]. Health care processes have changed due to the introduction of digital health care applications, especially video and e-consultations. New guidelines were created remarkably quickly, and existing ones were adapted, thanks partly to good support from professional organisations. Thus, the lesson learned is to retain the positive aspects of these adjustments and avoid the negative consequences.

Triage in GP practices has been introduced on a larger scale for safety and infection prevention reasons. No comparable pre-pandemic figures are available, but triage was likely deployed more thoroughly during the pandemic. No patient came to the practice without the reason and the urgency of each visit was discussed with the practice assistant, the practice nurse or the GP. Patients did not always appreciate that. Stronger triage can create extra barriers for people who have difficulty expressing themselves – due to language difficulties or low health literacy. This may have contributed to delays in seeking care for some categories of patients. Also, video consultations do not work well for all patients or is appreciated by them. If patients cannot withdraw during the video consultation, this can lead to privacy issues. Moreover, for video consultations, just as with triage, not all patients are equally skilled with the technology and articulating their care needs over this medium.

Many GPs indicated they sometimes saw patients late, because they did not come to the practice or contacted them earlier. That will certainly be experienced as a problem, but the question is whether practices could have influenced this and how. Half of the practices used their knowledge of their own patient population to approach certain patients actively. The corona files on the website of NHG have also responded to this, providing regularly updated information and recommendations, based on advancing insight and experiences in the field. This went together with the mutually coordinated information from the National Association of GPs (LHV) and the umbrella organisation for health centres, GP out-of-hours organisations, and GP care groups (InEen).

The collaboration within practices and with neighbouring practices to deal with problems of absenteeism, reflects one of the recently reassessed core values ​​of the profession: mutual cooperation in primary care [23]. The time freed up because of reduced numbers of patients visiting the practice, has led in some practices to more supervised triage and perhaps also more support from the practice staff in general.

The results of this descriptive study lead to several suggestions for similar situations in the future.

- GP organisations have an important role in the providing information; it is useful to monitor the experiences of GPs so that recommendations and guidelines can be adapted quickly if circumstances require.
- It is important to give practice staff the feeling that they are supported in providing good and safe care, and similarly to give patients the feeling that they can visit the practice safely and on time. This requires risk assessments based on knowledge about the practice population and the specific context of the patients (by using a person-oriented approach) [24].
- Patients must be actively informed about the accessibility of care in understandable language [25]. It is important to investigate the long-term consequences for, for example, patients who (for whatever reason) have not been seen or have only been seen late, and thus to reach a clear view of threats to patient safety and quality of care.

### Relevance to GPs in other countries

The specific GP system of the Netherlands may have helped Dutch practices to adapt during the pandemic. Tariffs for e-consultations were already existing. This saved much time that otherwise would have been taken by setting tariffs and rules. The list system with (nearly) all inhabitants registered to a practice of their choice, provided GPs with a well-defined patient population and, through their electronic files, with knowledge about patients. This has facilitated an outreaching approach. Also, support by the strong national college of GPs has supported practices in practical matters of, e.g., guidelines for infection prevention. Regular updates and intensive communication are a must.

These are all features of the Dutch GP care organisation that may not easily be translated to other health care systems, at least in the short run. However, evaluation of the response of primary care and general practice to the pandemic may provide arguments for changes in weaker primary care systems [26].

Finally, it is interesting to notice the small scale of Dutch GP practices that may have posed problems in coping with absenteeism. Good relations with neighbouring practices may have attenuated this and GPs in this study reported improved cooperation with neighbouring practices as an unintended side effect. Health care systems with larger practices, in terms of the number of GPs and other staff, may be more flexible in adapting to a crisis such as the COVID-19 pandemic. Also, the fragmentation of the Dutch health and social care system may have hampered a coordinated response. Countries with a more integrated system of primary care, public health, home care and long-term care may be better able to provide a coordinated response. Payment per consultation and home visit, which is one part of the payment system in Dutch general practice, appeared to be a problem when the numbers of consultations decreased during the pandemic. Due to the restraint among patients to consult the practice, less accessibility became a result of practice level safety measures, although financial support was available to compensate at least partly for this [3]. Countries with salaried GPs may have been better able to cope with lower numbers of patient contacts, while those with only fee-for-service may have had more difficulty.

## Conclusion

Dutch general practices have responded to the COVID-19 pandemic with an adapted organisation of their practice, safety regulations and use of video consultations. Despite the absence of staff due to illness or quarantine, GP care was able to continue. Still, triage, remote care and lack of clarity about the accessibility of care may have led to incidents in patient safety, according to the GPs surveyed.

## Data Availability

The anonymized data is held at Ghent University and is available to participating partners for further analysis upon signing an appropriate data transfer agreement.

## Abbreviations

EFPC: European Forum for Primary Care
EGPRN: European General Practice Research Network
EQuiP: European Society for Quality and Patient Safety in General Practice/Family Medicine
GPs: General practitioners
InEen: The Dutch umbrella organisation for health centres, GP out-of-hours organisations, and GP care groups
LHV: National Association of General Practitioners
NHG: Dutch College of General Practitioners
RIVM: National Institute of Public Health
